# Estimation of chimpanzee age based on DNA methylation

**DOI:** 10.1038/s41598-018-28318-9

**Published:** 2018-07-03

**Authors:** Hideyuki Ito, Toshifumi Udono, Satoshi Hirata, Miho Inoue-Murayama

**Affiliations:** 1 Wildlife Research Center, Kyoto University, Kyoto, Japan; 2 Kyoto City Zoo, Kyoto, Japan

## Abstract

In wild animal conservation, knowing the age of an individual animal is extremely beneficial. However, estimating the age is difficult for many species. Recently, epigenetics-based methods of estimating age have been reported. These studies were predominantly on humans with few reports on other animals, especially wild animals. In the present study, a chimpanzee (*Pan troglodytes*) age prediction model was developed based on the *ELOVL2*, *CCDC102B*, and *ZNF423* genes that may also have application in human age prediction. Pyrosequencing was used to measure methylation in 20 chimpanzee blood samples and correlation between age and methylation status was calculated. Age and methylation of sites in *ELOVL2* and *CCDC102B* were significantly correlated and an age prediction model was created using these genes. In the regression equation using only *ELOVL2*, the highest correlation coefficient was 0.741, with a mean absolute deviation (MAD) of 5.41, compared with the combination of *ELOVL2* and *CCDC102B*, where the highest correlation coefficient was 0.742 and the MAD was 5.41. Although larger MADs were observed in chimpanzees than in humans based on these genes, the results indicate the feasibility of estimating chimpanzee age using DNA methylation, and can have implications in understanding the ecology of chimpanzees and chimpanzee conservation.

## Introduction

Age is critical information to know about an animal because various biological characteristics change with aging, including those affecting biological status of animals (e.g. reproductive traits^1–5^ and mortality^6–8^). Animal age is a significant factor in the animal population as age distribution within a population can significantly influence maintenance of the population. Therefore, being able to estimate individual age is essential to understanding the life history of animals. Although many methods have been reported for age estimation of animals (e.g. based on incremental lines in teeth, tooth condition^9^, pulp/tooth area ratios^10^, and skeleton ossification^11,12^), many species have no external features that allow age to be estimated reliably in a noninvasive manner. Recently, various molecular genetics-based methods (e.g. mutation accumulation in mitochondrial or nuclear DNA, changes in mitochondrial DNA copy number, telomere length, and DNA methylation) of estimating age have been reported^13,14^.

DNA methylation is a form of epigenetic modification and cytosine-5 methylation of CpG dinucleotides is the best-studied epigenetic modification^15^. The regions upstream of many genes contain CpG dinucleotide clusters (i.e. CpG islands), where DNA methylation of a gene’s promoter can repress gene expression^15^. Many studies have reported correlations between DNA methylation at CpG islands and age^16–19^. There are two types of age-related DNA methylation: epigenetic drift and epigenetic clock^15,20,21^. While epigenetic drift has an inconsistent relationship with age across individuals, epigenetic clock is consistent with age across individuals^15,21^. Therefore, epigenetic clock type is more useful when estimating age and has previously been used to estimate age in humans and other animals^16–19,21^.

Chimpanzees (*Pan troglodytes*) are the most abundant of the great ape species. However, due to poaching, infectious disease, and loss of habitat due to the expansion of human activities, this species has experienced a significant reduction in population. It is currently considered “Endangered” by the International Union for Conservation of Nature and is listed in appendix I of the Convention on International Trade in Endangered Species of Wild Fauna and Flora^22^. Although age has been estimated in chimpanzees using teeth and bones as for other animals, this can be difficult in matured individuals. Therefore, an accurate method of estimating age in adult chimpanzees need to be developed. Previous analysis of DNA methylation in 6 chimpanzees suggests this may be a possible basis for age estimation of chimpanzees^20^.

In the present study, we analysed DNA methylation of three genes in the epigenetic clock region of humans^19^, *ELOVL2* (elongation of very long chain fatty acids protein 2), *CDCC102B* (coiled-coil domain-containing protein 102B), and *ZNF423* (zinc-finger protein 423), to develop an accurate age prediction model for chimpanzees.

## Results

In order to develop an age prediction model for chimpanzees, DNA methylation was analysed for 20 DNA samples obtained from chimpanzees aged 2 to 39 years. Fourteen CpG methylation sites were evaluated, where 9 were in *ELOVL2*, 2 were in *CDCC102B*, and 3 CpG were in *ZNF423* (Table 1). Correlations between the amount of DNA methylation at each site and chronological age were calculated in the form correlation coefficients (Table 1). It was found 5 CpG sites in the three genes, where 4 were in *ELOVL2* and 1 was in *CCDC102B*, met the criteria of a correlation coefficient of r >0.5. The strongest correlation was observed for methylation at position CpG7 in *ELOVL2* (*r* = 0.652), which explained 43% of the age variance (R2 = 0.426). The second strongest correlation was at position CpG3 in *ELOVL2* (*r* = 0.585) followed by CpG1 in *ELOVL2* (*r* = 0.556). In *CCDC102B*, correlations for age with CpG1 and CpG2 were −0.507 and −0.419, respectively. Methylation at all CpG sites in *ZNF423* failed to meet our criteria of a correlation coefficient of >0.5. These sites were not further analysed. Age prediction models were developed using multivariate linear regression based on the CpG sites assessed in this study (Table 2). In the simplest and most cost-effective age prediction model, which combined multiple methylation sites in the *ELOVL2* gene, the correlation coefficients ranged from 0.652 (only methylation site CpG7) to 0.741 (combination of four methylation sites). The mean absolute deviations (MADs) calculated for the single-gene model ranged from 5.41 (combined four methylation sites) to 6.79 years (CpG7). In addition, two multivariate regression models combining CpG islands from both *ELOVL2* and *CDCC102B* were assessed. In the first, the CpG site that correlated strongest with age was selected from each gene. In the second, all CpG cites with correlation coefficients of >0.5 were combined. The correlations from the first and second models were 0.675 and 0.741 with MADs of 6.18 and 5.42, respectively.

**Table 1:** Correlation between Age and Methylation Status at Each Methylation Site.

Gene	Methylation sites	Correlation coefficient	Position in *Pan_troglodytes-2.1.4*
*ELOVL2*	CpG-1	0.556	11130889
	CpG-2	0.490	11130890
	CpG-3	0.585	11130893
	CpG-4	0.296	11130899
	CpG-5	0.379	11130901
	CpG-6	0.524	11130903
	CpG-7	0.652	11130906
	CpG-8	0.449	11130914
	CpG-9	0.339	11130920
*CCDC102B*	CpG-1	−0.507	64948171
	CpG-2	−0.419	64948198
*ZNF423*	CpG-1	0.191	48479893
	CpG-2	0.024	48479897
	CpG-3	0.046	48479908

**Table 2:** Validation of Age Prediction using Multiple DNA Methylation Sites.

Regression model	Methylation sites	Correlation coefficient	Mean Absolute Deviation	*p* - value
Single gene	*ELOVL2 (CpG-7, 3)*	0.669	6.69	0.0065
	*ELOVL2 (CpG-7, 3, 1)*	0.731	5.43	0.0056
	*ELOVL2 (CpG-7, 3, 1, 6)*	0.741	5.41	0.0131
Multiple genes	*ELOVL2 CpG-7* + *CCDC102B CpG-1*	0.675	6.18	0.0057
	*ELOVL2 (CpG-7,3,1,6)* + *CCDC102B CpG-1*	0.741	5.42	0.0318

## Discussion

Wild animal age assessment is very important for studies about life history^6,8^. However, this can be difficult and, depending on the species, can be extremely laborious and/or obtained only by field studies over a long period. DNA methylation is the most widely studied form of epigenetic modification in relation to aging. Pyrosequencing can measure methylation at different CpG sites with high accuracy. A population of individuals of known age is required when creating an age prediction model. Therefore, a captive population of animals of a known age would be particularly useful, especially for long-lived species, because a longer time period is required to determine the age of individuals in a wild population. However, there have been only a few reports on age prediction using DNA methylation in animals outside of humans (e.g. dogs^16,21^, wolf^21^, humpback whales^17^, and chimpanzees^20^) to date.

In the present study, the correlation between average methylation status for each gene assessed and age was significant only for *ELOVL2* (*p* = 0.0021). No significant correlation was found for the other two genes. When assessing each individual methylation site, the methylation status of five sites in two genes, *ELOVL2* and *CCDC102B*, was significantly correlated with age. With age, the methylation status increased for *ELOVL2*, but decreased for *CCDC102B*, which is similar to the trend observed in humans^19^. However, while the methylation of these genes for most of the eight chimpanzees sampled at the two timepoints (recent and 20 years ago) followed this trend, there were a few individuals whose methylation displayed the opposite phenotype (Fig. S1). It has been reported that methylation can be altered by factors such as cancers and Alzheimer’s disease^23–25^, so, individuals with different tendencies might have been affected by such factors. However, there was no evidence of any disorders, pregnancy, transfer, etc., in our records. In order to verify methylation of certain genes as a useful marker for assessing age, a larger number of samples needs to be assessed in future work.

The life expectancy of chimpanzees in the wild at Ngogo, Kibale National Park, Uganda averages 32.8 years^26^, and chimpanzees are considered to have a maximum longevity of over 60 years in the wild based on a study sampling several countries^27^. The lifespan of chimpanzees in captivity is believed to be longer than that in the wild. The oldest individual in this study was 39 years old, and the number of old-age individuals analysed was not optimal. Age prediction using DNA methylation deviates more from the predicted age as the actual age increases^19^. Therefore, assessment of elderly individuals is necessary to verify the usefulness of age prediction models. Moreover, the MADs in this study ranged from 5.42 to 6.79 years. Considering that the chimpanzee lifespan is about 50 years, these deviations represent more than 10% of the chimpanzee lifespan. These values are higher than those typically observed in humans (e.g. 3.156^19^ and 3.07 years^28^). While all the models developed in the present study were significant, the MADs were still relatively large. Also, as the size of the samples was relatively small, further investigations using bigger samples are needed to increase confidence in these findings. Therefore, it is necessary to increase the number of individuals or genes surveyed and develop a more appropriate model in the future. Analysis of age based on methylation of a single locus had the most significant, but the lowest correlation coefficient. Conversely, analysis of methylation at multiple loci yielded a relatively low *p* value but a high correlation coefficient. Therefore, it is essential to identify a modest number of methylation sites to combine for use in age prediction.

Furthermore, only blood samples were used in this study and it is difficult to collect blood from wild individuals and desirable to use samples collected using less invasive methods in captive individuals. Therefore, the development of age prediction methods using low or non-invasive sampling (such as using hairs and faeces) is required. DNA methylation is more stable than other biomarkers and therefore may be more suitable for age estimation, especially in degraded biological samples. While epigenetic clock gene methylation is similar across individuals, there can be tissue specificity^15^. In humans, it has been reported determining age from saliva is equally accurate to using blood. For example, Hong *et al*.^29^ generated a multiplex age prediction model using saliva exhibiting MADs of 3.1 to 3.2 years. To predict age using DNA methylation with samples collected in a non-invasive manner, it is important to survey different types of chimpanzee samples.

In this study, we estimated the age of chimpanzees by measuring methylation with pyrosequencing. Two of the 3 tested genes, *ELOVL2* and *CCDC102B*, have the potential to be useful for estimating age. Age prediction using multivariate regression based on methylation provided a correlation coefficient of 0.741 and a MAD of 5.42 years. This supports the potential use of pyrosequencing in predicting age based on DNA methylation in chimpanzees. Being able to accurately estimate age based on DNA methylation will contribute to understanding the ecology of chimpanzees and would be useful in conservation efforts.

## Methods

### Sample collection

This study was conducted in strict accordance with the guidelines for the ethics of animal research established by the Kyoto City Zoo and the Wildlife Research Center (WRC) of Kyoto University, Japan. All animal experiments of study were approved by the animal experiment committee of WRC, Kyoto University (Approval No: WRC-2017-006A). Twenty blood samples were obtained as byproducts of health examinations from 12 chimpanzees, where 12 samples were collected from 2008–2009 and the remaining 8 were from 1998 (Table 3). Animals with GAIN ID: 131, 132, 146, 159, and 268 were wild-derived and others were bred in captivity in the Kumamoto Sanctuary at the Wildlife Research Center of Kyoto University. Further information on each individual chimpanzee in this study can be obtained from the Great Ape Information Network (https://shigen.nig.ac.jp/gain/top.jsp). Collected white blood cells were stored at −20 °C. DNA was extracted using the QIAGEN DNeasy Blood and Tissue Kit (Qiagen, Germany). The quality and quantity of the extracted genomic DNA were assessed using an ND-1000 spectrophotometer (Nanodrop Technologies, Wilmington, DE).

**Table 3:** Chimpanzee Blood Samples.

Sample number	Gain ID number*	Age (years)	Year Collected
1	258	2	1988
2	268	13	1988
3	201	5	1988
4	262	2	1988
5	159	10	1988
6	132	18	1988
7	146	11	1988
8	131	14	1988
9	549	12	2008
10	567	11	2008
11	258	22	2008
12	268	33	2008
13	201	25	2008
14	262	22	2008
15	159	30	2008
16	556	12	2009
17	132	39	2009
18	146	32	2009
19	131	35	2009
20	600	10	2009

*Great Ape Information Network (https://shigen.nig.ac.jp/gain/index.jsp).

### Pyrosequencing

Candidate age-correlated markers were evaluated by pyrosequencing. Pyrosequencing was performed using Alliance Biosystems (Japan). Briefly, bisulfite conversion was performed on 300 ng of total DNA from each sample using the EZ DNA Methylation Lightning kit (Zymo Research, Irvine, CA). For pyrosequencing, forward, reverse, and sequencing primers, which are listed in Table 4, were designed using PyroMark Assay Design version 2.0.1.15 (Qiagen) based on the chimpanzee genome (*Pan_troglodytes*−2.1.4). To perform PCR, 2.5 μL of bisulfite-converted DNA, 10 μL 2 × TOPsimple^TM^ DyeMIX-HOT (Enzynomics, Inc., Korea), 10 pmol of each primer, and distilled water were combined for a total reaction volume of 20 μL. PCR cycling was conducted in a Veriti Thermal Cycler (Applied Biosystems, Waltham, MA) with the following conditions: 95 °C for 10 min, then 45 cycles of 95 °C for 30 s, 54 °C for 30 s, and 72 °C for 30 s, and finally extension at 72 °C for 5 min. PCR product (18 μL) was immobilized on 3 μL of Streptavidin Sepharose High Performance beads (GE Healthcare Bio-Sciences, Uppsala, Sweden) and annealed with the sequencing primer for 2 min at 80 °C. Pyrosequencing was carried out using the PyroMark ID system (Qiagen) with the Pyro Gold reagent kit (Qiagen) according to the manufacturer’s instructions. After pyrosequencing, the percent methylation was calculated using PyroMark ID software and the results were displayed as a pyrogram with methylation values for each CpG site.

**Table 4:** Primer List.

Gene	Primer sequence	
*ELOVL2*	Forward	GGGAGGGGAGTAGGGTAA
	Reverse	CCATTTCCCCCTAATATATACTTCAA
	Sequencing*	GGGAGGAGATTTGTAGGTTT
*CCDC102B*	Forward	TTTTGGTTTTGAATGTTGTTGAGAGAGG
	Reverse	TACCCTTCTCTAAAAATAACTATCCTT
	Sequencing*	GAGAGGGGAATGTTTG
*ZNF423*	Forward	GTTTAGTAGAGGGAGATAGTAGATGTGT
	Reverse	AATCCCTATCTCTAATTAAACCATTACC
	Sequencing*	AGGAGTGTTGAGGTAGTG

*Primer for pyrosequencing.

### Development of age prediction model

Correlations between age and methylation status of the 14 tested CpG sites were examined using linear regression. Initially, a bivariate correlation was determined for each CpG site. For each methylation site, a correlation coefficient with age was calculated. Only methylation sites with a correlation coefficient of 0.5 or more were used for age prediction. Three regression equation models were considered for age prediction. The first model included DNA methylation sites in only a single gene. This model was the simplest and most cost-effective. The other two models were based on DNA methylation sites in multiple genes. The second model was a regression equation based on one methylation site with the highest regression coefficient for each gene. The third model involved a regression equation combining all 5 methylation site sites with a correlation coefficient of 0.5 or more.

## Electronic supplementary material

Figure S1. Alterations in Methylation Over 20 Years at Each DNA Methylation Site

## References

[1] Charpentier MJ, Tung J, Altmann J, Alberts SC (2008). Age at maturity in wild baboons: genetic, environmental and demographic influences. Mol Ecol..

[2] Tkadlec E, Zejda J (1998). Density-dependent life histories in female bank voles from fluctuating populations. J. Anim Ecol..

[3] Nussey DH (2008). Testing for genetic trade-offs between early- and late-life reproduction in a wild red deer population. Proc Biol Sci..

[4] Bowen WD, Iverson SJ, McMillan JI, Boness DJ (2006). Reproductive performance in grey seals: age-related improvement and senescence in a capital breeder. J. Anim Ecol..

[5] Atsalis S, Videan E (2009). Reproductive aging in captive and wild common chimpanzees: factors influencing the rate of follicular depletion. Am J Primatol..

[6] Low M, Part T (2009). Patterns of mortality for each life-history stage in a population of the endangered New Zealand stitchbird. J. Anim Ecol..

[7] Nussey DH, Froy H, Lemaitre JF, Gaillard JM, Austad SN (2013). Senescence in natural populations of animals: widespread evidence and its implications for bio-gerontology. Ageing Res Rev..

[8] Toigo C (2007). Sex- and age-specific survival of the highly dimorphic Alpine Ibex: evidence for a conservative life-history tactic. J. Anim Ecol..

[9] Chevallier, C., Gauthier, G. & Berteaux, D. Age Estimation of live Arcticfoxes *Vulpes lagopus* based on teeth condition. *Wildl**Biol*, wlb.00304 (2017).

[10] White PA (2016). Age Estimation of African Lions *Panthera leo* by Ratio of Tooth Areas. PLoS ONE..

[11] Serrano E, Gállego L, Pérez JM (2004). Ossification of the appendicular skeleton in the Spanish Ibex *Capra pyrenaica* Schinz, 1838 (Artiodactyla: Bovidae), with regard to determination of age. Anatomia, Histologia, Embryologia..

[12] Siegal-Willott J, Isaza R, Johnson R, Blaik M (2008). Distal limb radiography, ossification, and growth plate closure in the juvenile Asian elephant (*Elephas maximus*). J. Zoo Wildl Med..

[13] Jarman SN (2015). Molecular biomarkers for chronological age in animal ecology. Mol Ecol..

[14] Xia X, Chen W, McDermott J, Han JJ (2017). Molecular and phenotypic biomarkers of aging. F1000Res..

[15] Jones MJ, Goodman SJ, Kobor MS (2015). DNA methylation and healthy human aging. Aging Cell..

[16] Ito G, Yoshimura K, Momoi Y (2017). Analysis of DNA methylation of potential age-related methylation sites in canine peripheral blood leukocytes. J. Vet Med Sci..

[17] Polanowski AM, Robbins J, Chandler D, Jarman SN (2014). Epigenetic estimation of age in humpback whales. Mol Ecol Resour..

[18] Garagnani P (2012). Methylation of ELOVL2 gene as a new epigenetic marker of age. Aging Cell..

[19] Park JL (2016). Identification and evaluation of age-correlated DNA methylation markers for forensic use. Forensic Sci Int Genet..

[20] Horvath S (2013). DNA methylation age of human tissues and cell types. Genome Biol..

[21] Thompson MJ, vonHoldt B, Horvath S, Pellegrini M (2017). An epigenetic aging clock for dogs and wolves. Aging..

[22] Humle, T., Maisels, F., Oates, J. F., Plumptre, A. & Williamson, E. A. *Pan troglodytes*, http://www.iucnredlist.org (2016).

[23] Baylin SB (2012). The cancer epigenome: Its origins, contributions to tumorigenesis, and translational implications. Proc Am Thorac Soc..

[24] Baylin SB, Jones PA (2011). A decade of exploring the cancer epigenome - biological and translational implications. Nat Rev Cancer..

[25] Roubroeks JAY, Smith RG, van den Hove DLA, Lunnon K (2017). Epigenetics and DNA methylomic profiling in Alzheimer’s disease and other neurodegenerative diseases. J. Neurochem..

[26] Wood BM, Watts DP, Mitani JC, Langergraber KE (2017). Favorable ecological circumstances promote life expectancy in chimpanzees similar to that of human hunter-gatherers. J. Hum Evol..

[27] Emery Thompson M (2007). Aging and fertility patterns in wild chimpanzees provide insights into the evolution of menopause. Curr Biol..

[28] Freire-Aradas A (2016). Development of a methylation marker set for forensic age estimation using analysis of public methylation data and the Agena Bioscience EpiTYPER system. Forensic Sci Int Genet..

[29] Hong SR (2017). DNA methylation-based age prediction from saliva: High age predictability by combination of 7 CpG markers. Forensic Sci Int Genet..

