# Supplementary material for: Estimation of chimpanzee age based on DNA methylation
Source: Sci Rep. 2018 Jul 3;8:9998. doi: 10.1038/s41598-018-28318-9 (PMC6030051; doi:10.1038/s41598-018-28318-9)
Supplement: Supplementary file 1 — Figure S1. Alterations in Methylation Over 20 Years at Each DNA Methylation Site [file 41598_2018_28318_MOESM1_ESM.pdf]

Supplementary information

Title: Estimation of chimpanzee age based on DNA methylation

Hideyuki Ito[1,2], Toshifumi Udono[1], Satoshi Hirata[1], Miho Inoue-Murayama[1*]

[1]Wildlife Research Center, Kyoto University, Kyoto, Japan

[2]Kyoto City Zoo, Kyoto, Japan

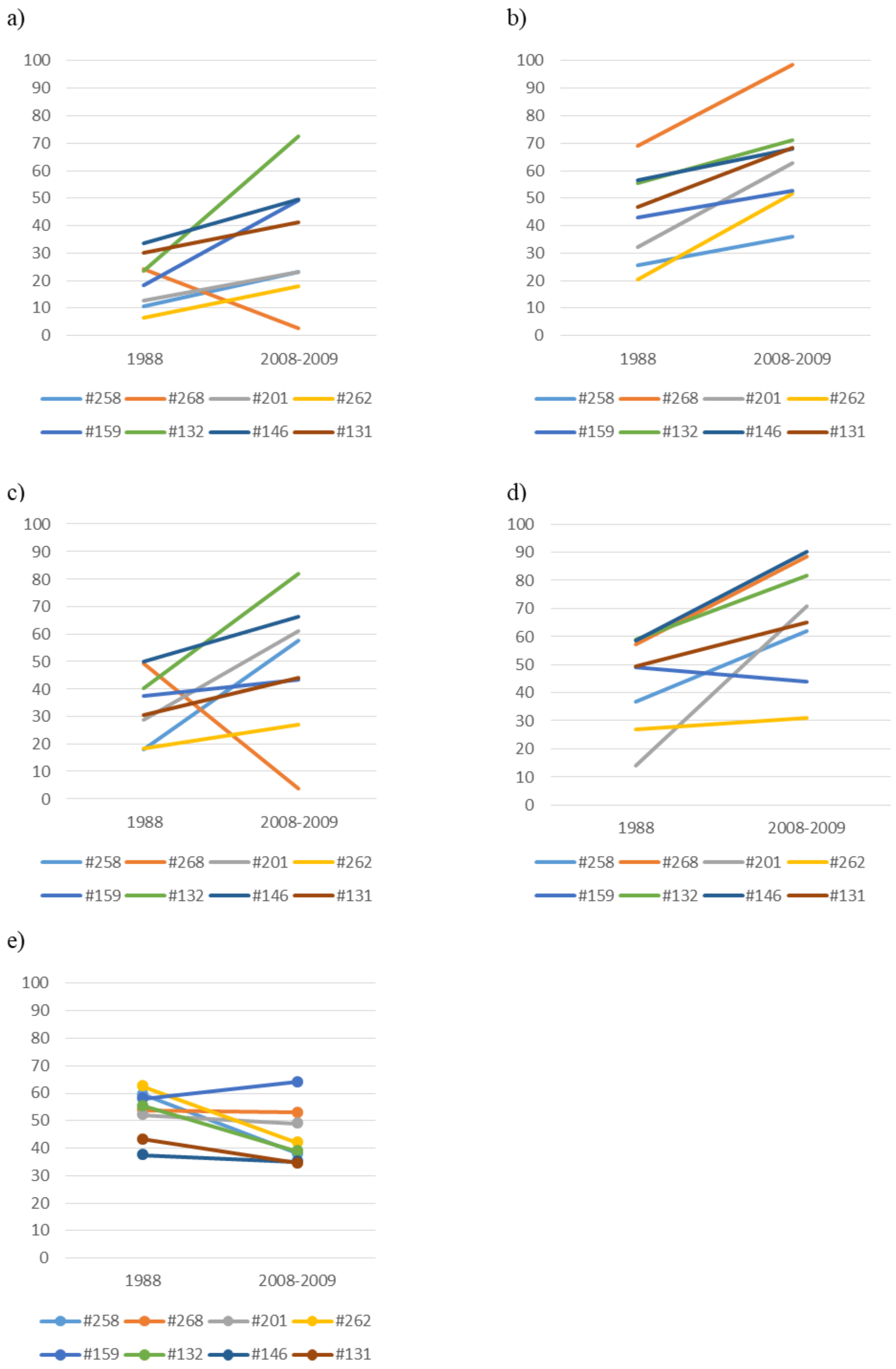

Figure S1. Alterations in Methylation Over 20 Years at Each DNA Methylation Site

Numbers is vertical axis indicate percentage of methylation. #number indicates GAIN ID number. (a) *ELOVL2*-CpG1, (b) *ELOVL2*-CpG3, (c) *ELOVL2*-CpG6, (d) *ELOVL2*-CpG7, and (e) *CCDC102B*-CpG1
